# The use of UK primary care databases in health technology assessments carried out by the National Institute for health and care excellence (NICE)

**DOI:** 10.1186/s12913-020-05529-3

**Published:** 2020-07-22

**Authors:** Thomas P. Leahy, Sreeram Ramagopalan, Cormac Sammon

**Affiliations:** 1 PHMR Ltd., Berkeley Works, Berkley Grove, London, NW1 8XY UK; 2 Centre for Observational Research and Data Sciences, Bristol-Myers Squibb, Uxbridge, U88 1DH UK

**Keywords:** CPRD, Appraisals, Guidance

## Abstract

**Background:**

Real world evidence (RWE) is becoming more frequently used in technology appraisals (TAs). This study sought to explore the use and acceptance of evidence from primary care databases, a key source of RWE in the UK, in National Institute for Health and Care Excellence (NICE) technology assessments and to provide recommendations regarding their use in future submissions.

**Methods:**

A keyword search was conducted relating to the main primary care databases in the UK on the NICE website. All NICE TAs identified through this search were screened, assessed for duplication and information on the data source and the way the data was used in the submission were extracted. Comments by the evidence review group (ERG) and the appraisal committee were also extracted and reviewed. All data extraction was performed by two independent reviewers and all decisions were reached by consensus with an additional third reviewer.

**Results:**

A total of 52 NICE TAs were identified, 47 used the General Practice Research Database /Clinical Practice Research Datalink (GPRD/CPRD) database, 10 used The Health Improvement Network (THIN) database and 3 used the QResearch databases. Data from primary care databases were used to support arguments regarding clinical need and current treatment in 33 NICE TAs while 36 were used to inform input parameters for economic models. The databases were sometimes used for more than one purpose. The data from the three data sources were generally well received by the ERGs/committees. Criticisms of the data typically occurred where the results had been repurposed from a published study or had not been applied appropriately.

**Conclusions:**

The potential of UK primary care databases in NICE submissions is increasingly being realised, particularly in informing the parameters of economic models. Purpose conducted studies are less likely to receive criticism from ERGs/committees, particularly when providing clinical input into cost effectiveness models.

## Background

The National Institute for Health and Care Excellence [1] is a UK national body aimed at improving outcomes for patients using public health and social care services [1]. One of its several duties is to assess the clinical and cost effectiveness of health technologies including but not limited to new pharmaceutical products. This type of assessment by NICE is called a technology appraisal [2]. As of April 2019, NICE had published 77 TAs for 2017/2018 and a total of 572 TAs published since 2000 [3].

For NICE to make appraisal decisions it must consider all available evidence including comparison with relevant alternative treatments. This information is unlikely to be available from a single source and evidence generation from alternative sources of data including real-world evidence is required to obtain a full overview of cost effectiveness [4]. Real World Evidence (RWE) [5], that is, evidence obtained outside the context of a randomised controlled trial (RCT), enables a more robust critical assessment of technologies and can validate whether the study population and clinical context of a RCT is reflective of clinical practice [6]. As such, the interest of RWE in the assessment of health technologies and in TA submissions are becoming increasingly prevalent [7, 8]. This increasing interest is clearly demonstrated with large amounts of funding through various sources including the Innovative Medicines Initiative going into a European wide consortium that aims to show the value of RWE in healthcare decision making.

Electronic health records (EHRs) are a systematic collection of individual patient data generally collected at source and stored digitally. This data is then used both to improve patient care and improve patient outcomes. Some of this data is anonymised and made available for researchers to study. One of the most comprehensive systems for collecting patient’s data globally is in the UK, the Clinical Practice Research Datalink (CPRD). The CPRD collects data including demographics, diagnoses, symptoms, prescriptions, tests and immunisations from UK GP practices [2]. As of mid-2013, the CPRD held 11.3million patient records of which 4.4million were still alive and currently registered with a CPRD practice, this represented approximately 6.9% of the total UK population [2]. Other primary care databases include The Health Improvement Network (THIN) [9] and QResearch [10]. Linkage of these primary care databases with databases of secondary care data (Hospital Episode Statistics) and disease registries is also increasingly common.

Given the breadth of data available in UK primary care databases, and the possibility of linkage with additional data sources, they offer great potential to be used to inform multiple aspects of a NICE TA, including the epidemiology of conditions, the nature of their current management and the clinical and economic outcomes associated with them. In order to fully understand their potential, it is important for investigators to understand their use in NICE TAs to date and how the data presented has been received. To this end, this study reviews NICE TA submissions that have utilised primary care databases in the UK and reveals for which purpose the databases were used and how they were received by the ERG and appraisal committees.

## Methods

### Search strategy

The search strategy adopted followed the Preferred Reporting Items for Systematic Reviews and Meta-Analyses (PRISMA) guidelines [11]. The PRISMA 2009 checklist is used and applicable items included [12].

There are three large primary care databases generally used in NICE TA submissions they are the General Practice Research Database (GPRD) which in 2012 was renamed the Clinical Practice Research Datalink (CPRD), the Health Improvement Network (THIN) database and QResearch. Each of these databases are searched for within NICE TA submissions.

**Fig. 1:**
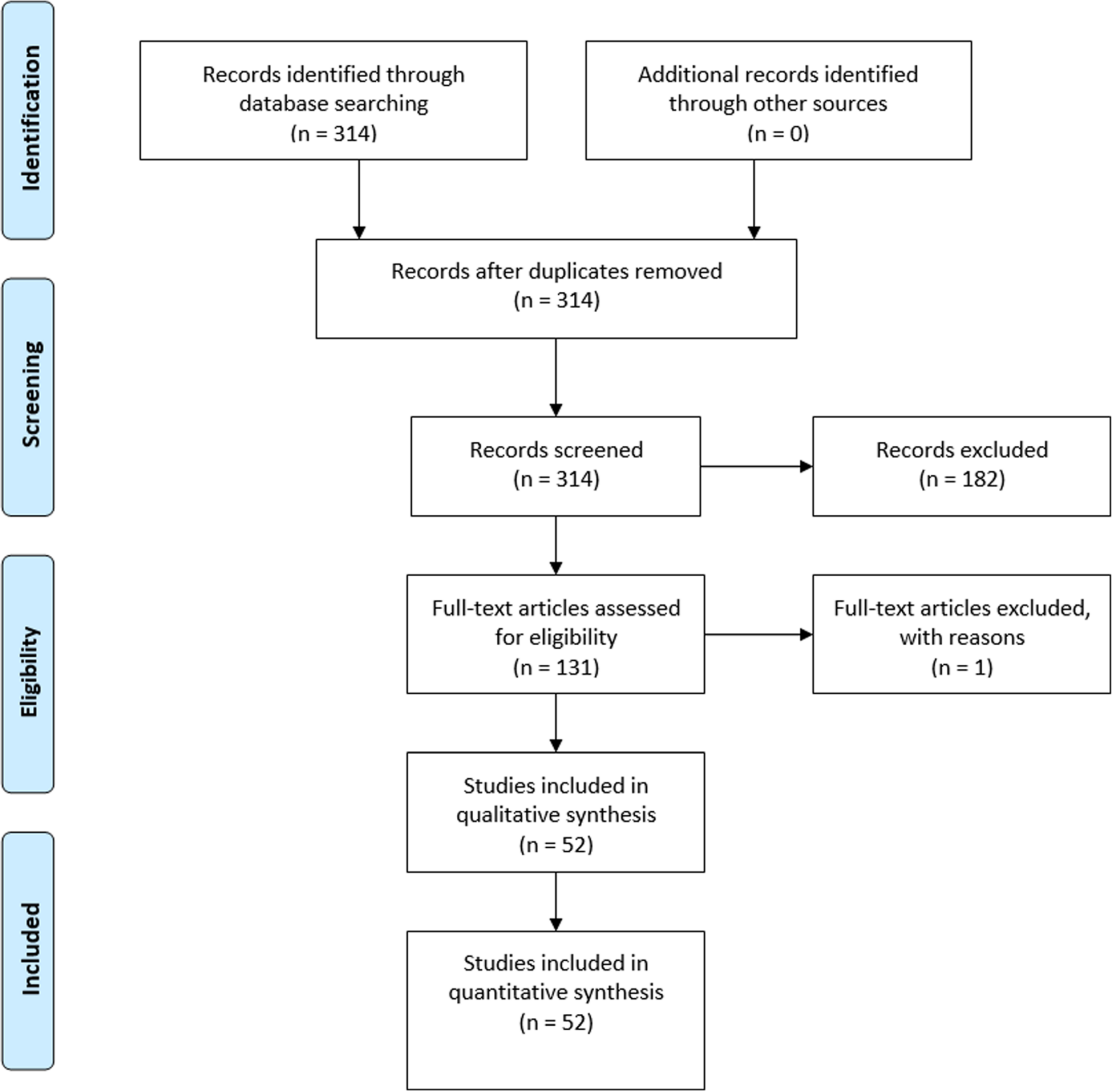
PRISMA flowchart for the systematic review of documents retrieved by search. The flowchart template is taken from the PRISMA website (http://prisma-statement.org/PRISMAStatement/FlowDiagram.aspx) [12]. Of the total documents retrieved, 182 were excluded as they were unrelated to TAs and a further document was excluded as this related to a TA that was withdrawn. The remaining 131 documents were grouped into 52 TAs

All NICE TAs that used one of the three primary care databases of interest were identified using a systematic search using a Google site search on the NICE website under the guidance section, “site:https://www.nice.org.uk/guidance/”. Within this section of the NICE website, the following keywords or phrases were used to identify relevant documents, “clinical practice research datalink”, “clinical practice research database”, “cprd”, “general practice research datalink”, “general practice research database”, “gprd”, “the health improvement network”, “THIN database” and “qresearch”. A record of all search results was maintained. Of the results returned, all documents not relating to a technology appraisal, determined through the document URL, were excluded. The remaining relevant documents were then grouped by TA number which is a unique identifier of each NICE TA submission. Any TA submissions that were withdrawn were also excluded as well as any duplicate documents. The search included any results up to March 2019. A PRISMA flowchart (Fig. 1) representing the search and inclusion/exclusion criteria was generated in line with the 2009 PRISMA checklist [12].

### Data extraction

Two independent extractions of the data were conducted by two reviewers and any ambiguities were decided upon by a third independent reviewer. The data extracted for each TA submission was the TA number, the year of NICE publication of the TA, the indication of treatment, the primary care database used and whether a study was conducted using the database specifically for the purpose of the TA (‘for purpose’) or if it used results from an existing, published study that used one of the databases (‘referenced’), who it was used by (manufacturer/ERG), how the primary care data was used (to describe disease epidemiology, describe current treatment or to provide clinical input into cost effectiveness (CE) model) and any critiques by the ERG and appraisal committee with respect to the use of the primary care data and their sentiment towards its use (positive or negative). Additionally, the indications were grouped by disease area using the British National Formulary (BNF) chapters [13]. The extraction of data was not limited to the documents retrieved from the keyword search, rather the whole TA submission was considered.

### Analysis

The frequency of use of the three primary care databases in NICE TA submissions over time was calculated overall and separately for each of the three databases. For the overall figures the linear trend over time was also calculated. Percentages and counts are calculated and displayed for all TA submissions that used the primary care data for purpose compared to referenced and this is stratified by sentiment, either positive, negative or no comment. Similarly, percentages and counts are calculated and displayed for all TA submissions that used the primary care data to describe disease epidemiology, describe current treatment and to provide clinical input into cost effectiveness models and this is also stratified by sentiment. For the TAs that used the primary care data for clinical input into CE models, the same analysis is conducted and displayed to show the sentiment of the ERG and committee towards its use when it is for purpose compared to referenced. Additionally, a table is given displaying the counts and percentage of the total TAs used in each disease area [see Additional file 1].

## Results

### Search results

The search strategy returned 314 documents in the guidance section of the NICE website. Of these 314 documents, those not relating to technology appraisals were removed including diagnostic guidance’s, intervention procedures guidance’s and medical technology guidance’s. This resulted in 132 documents that were used in TA submissions and included at least one of the keywords or phrases used in the search. These 132 documents were then grouped by TA number. The TA number uniquely identifies TA submissions. This resulted in 53 unique NICE TA submissions between 2003 and (March) 2019. One of these was excluded as per the exclusion criteria since the TA submission was withdrawn, this resulted in a total of 52 NICE TA submissions. A subset of the TAs used multiple UK primary care databases in their submissions. This resulted in 47 submissions using the G/CPRD, 10 using THIN and 3 using the QResearch databases respectively. A list of the included NICE TA submissions in this analysis is provided in the additional materials [see Additional file 2].

### Primary care database usage

**Fig. 2:**
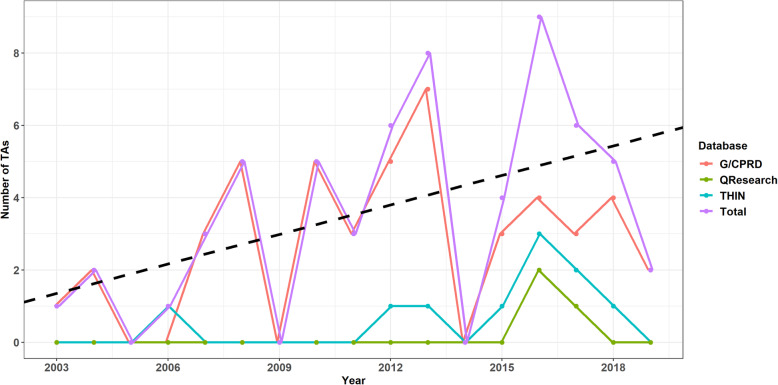
The number of NICE TA submissions using the G/CPRD, THIN and QResearch primary care databases shown by year of NICE publication. Additionally, the total is shown with its linear trend (black dashed line)

For the 52 NICE TA submissions that used a UK primary care database, a plot of the number of TAs published by NICE each year using a primary care database is shown in Fig. 2. The usage of primary care databases is becoming more prevalent in NICE TA submissions. The G/CPRD database is the most commonly used and accounts for the majority of total primary care database usage (~ 78%) compared to approximately 17 and 5% for THIN and QResearch respectively.

**Fig. 3:**
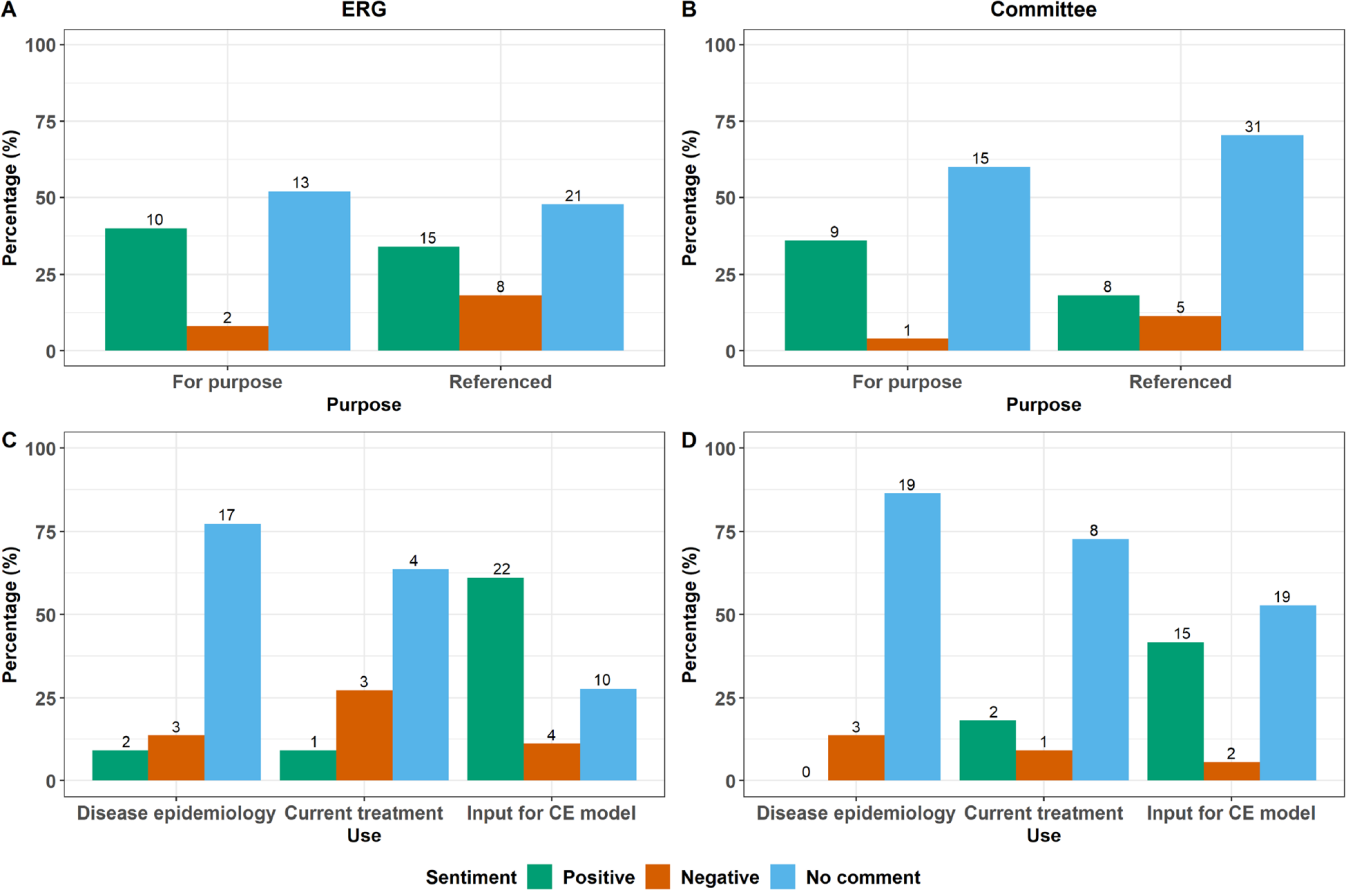
**a**, **b** Positive, negative sentiment or no comment with regards to using the primary care databases for purpose rather than referenced a previous study by the ERG and appraisal committee respectively. **c**, **d** Positive, negative sentiment or no comment with regards to using the primary care databases to describe disease epidemiology, describe current treatment and for clinical input into cost effectiveness models study by the ERG and appraisal committee respectively

From the numbers displayed in Fig. 3a and Fig. 3b, more NICE TA submissions have referenced the results of existing, published primary care database studies than have conducted studies using the databases specifically for the purpose of the TA submission. It was more likely that a NICE TA submission received a positive comment by both the ERG and appraisal committee if the usage of the primary care databases was for purpose rather than referencing another study (Fig. 3a and Fig. 3b). Negative comments were also more common for referenced usage than for purpose usage (Fig. 3a and Fig. 3b).

When the primary care databases were used to describe the disease epidemiology and to describe current treatment, comments were much less likely to be made by both the ERG and appraisal committees (Fig. 3c and Fig. 3d). When the databases were used to inform clinical input into CE models there was more of a response. There was a much more positive response to using primary care databases in informing clinical input into CE models both by the ERG and appraisal committees. Using the databases to inform clinical input to inform CE models accounted for approximately 52% of all usage compared to approximately 16 and 32% in describing current treatment and disease epidemiology respectively.

**Fig. 4:**
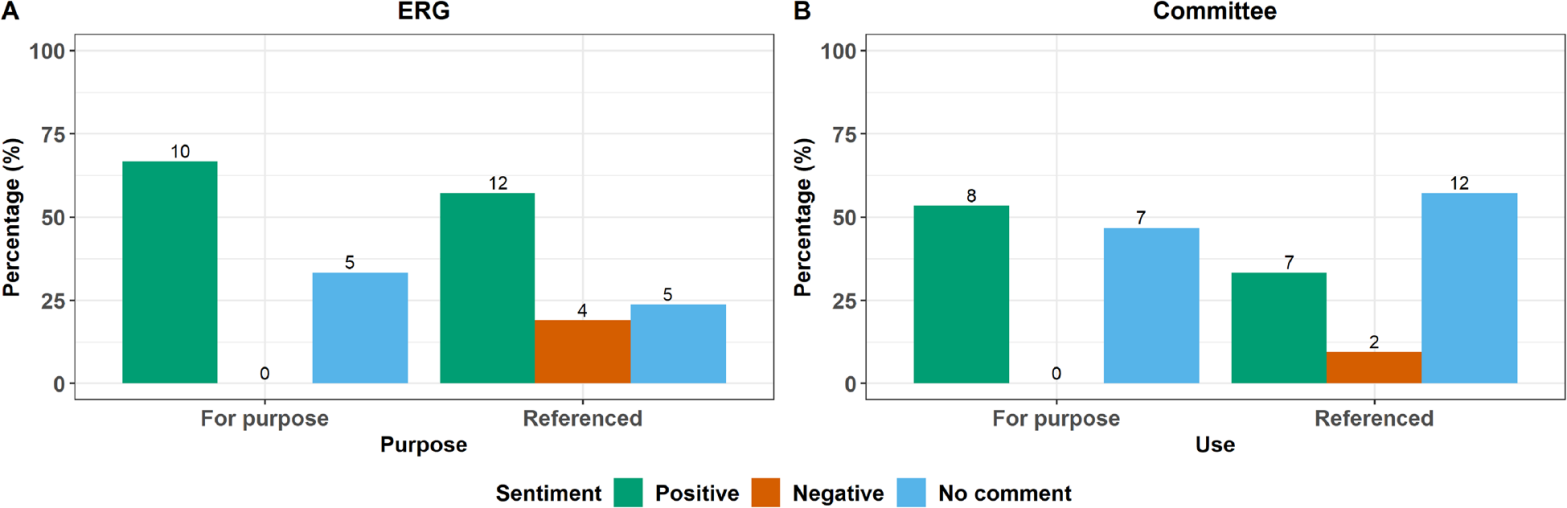
Positive, negative sentiment or no comment with regards to using the primary care databases to inform clinical input for cost effectiveness models for purpose compared to referencing a previous study by the ERG (**a**) and appraisal committee (**b**)

For the NICE TA submissions that used the primary care databases to provide clinical input into the CE models, a separate analysis of the ERG and appraisal committees sentiment towards studies used for purpose and those referenced is shown in Fig. 4. Additionally, some examples of positive and negative comments by the ERGs and appraisal committees toward the use of primary care databases in NICE TAs is given in Table 1. Repurposed studies were used more frequently in TA submissions to inform clinical input into CE models, accounting for approximately 58% of NICE TA submissions using primary care databases compared to approximately 42% using the databases specifically for the TA submission. Despite this, it is clear from Fig. 4, that when used to inform inputs to the economic model a positive comment was much more likely to be made by both the ERG and the appraisal committee if a study was conducted for the purpose of the TA submission than when the results of a published study were repurposed.

**Table 1:** Examples of positive and negative comments by the ERGs and appraisal committees

**Positive Comments**	
• “the General Practice Research Database data presented by the manufacturer have advantages over the [published] study in that they are more recent and therefore more reflective of the current UK atrial fibrillation population”	
• “annual mortality rates from the general UK population, adjusted for the increased risk of death in patients with moderate to severe psoriasis relative to matched controls based on the UK GPRD study … … the committee concluded that the company’s approach to modelling mortality reflected the best available evidence.”	
• “GPRD data were used as they may be more representative of the UK patient population than data from clinical trials. Use of GPRD data is also consistent with sources preferred in the NICE appraisals of ….”	
**Negative comments**	
• “The Committee was made aware of [CPRD] data indicating that acid-suppressive medication leads to a small increase in fracture risk …. The Committee was not persuaded by this evidence; it noted that the data are observational and have not been reported in full ….”	
• “… the ERG was concerned with the appropriateness of using CPRD data to estimate the resource use for the patient profiles observed in the trial because there were differences in the 2 populations.”	

## Discussion

This study has shown that the use of real-world evidence from primary care databases is becoming more prevalent in NICE TA submissions. Within these submissions, primary care databases have been used to describe the current epidemiology of a disease, to describe current treatment and/or to provide clinical input into CE models. When primary care databases were used to inform clinical input into CE models, they were much more positively received by both the ERG and appraisal committees when a study was carried out specifically for the purpose of deriving the input in question than when the results of a published study were repurposed.

The benefits of using primary care databases have been widely recognised, with the European Medicines Agency explicitly stating that RWE can support TAs by providing additional data to assess the proposed new technology [7, 8, 14]. To this end, NICE along with several other health technology assessment agencies, pharmaceutical manufactures, academic groups and regulatory bodies have developed a European consortium (https://rwe-navigator.eu/) to demonstrate the use of RWE and in the healthcare decision-making process [5]. One of the main outcomes of the RWE NAVIGATOR project was that all stakeholders including manufacturers, payers and TA bodies should consider routine use of RWE in the development and decision-making in TAs and that acceptability of RWE among stakeholders could be improved with the introduction of guidelines [15]. Our finding, that primary care databases are increasingly being used to inform NICE TAs, suggests that stakeholders are increasingly considering the potential of RWE in developing NICE submissions.

The lack of comments from the ERG and NICE committee when primary care databases were used to describe current disease epidemiology likely reflects the fact that these data were not incorporated into the CE model and therefore do not impact the observed ICERs. They are therefore of less relevance to the decision-making process and likely to receive less focus during review. However, in some cases it may also reflect an acceptance that these large longitudinal databases contain some of the best quality population level epidemiology data available for England, and that alternative, better quality sources are unlikely to be available. Similarly, the lack of comments observed when the databases were used to inform current treatment practices may reflect an acceptance that the databases are an accurate source of such data. Where criticism was observed it was typically due to the reuse of the results of existing studies or due to the manufacturers specifying an analysis not matching the decision problem, rather than an inherent limitation of the databases. However, in one case the ERG noted that the absence of secondary and tertiary care prescribing data limited the ability of the CPRD to provide data on current treatment practice. The lack of this data is a known limitation of these databases and should be considered in the design and interpretation of studies.

In contrast, the higher prevalence of comments observed when the databases were used to inform the clinical inputs into cost effectiveness models is likely to reflect the fact that any uncertainty in these inputs adds to the overall uncertainty surrounding the decision problem and that these inputs will therefore be subject to greater scrutiny by the ERG and committee. It is notable that when primary care data was used to inform CE models, all of the negative comments from the ERG and committee occurred when the data from a published study was repurposed for use in the NICE submissions. In such cases the ERG and committee expressed concerns about how the referenced data aligned with the decision problem based on the study population, the study period, the technologies studied and the study design. That analyses conducted specifically to inform the NICE submission were better-received by both ERGs and appraisal committees stands to reason, as the inputs are more likely to fit the parameters of the decision problem and the design of the economic model when developed for purpose. This suggests that where possible this approach should be taken when utilising primary care databases to support NICE TAs. However, we are aware that the timelines and costs of such studies can be somewhat prohibitive, with data acquisition costs for single studies typically exceeding £50,000 and considerable time required to prepare and analyse the data. In order to render the use of such databases in NICE submissions as cost effective as possible we therefore recommend that studies using the database are designed to inform as many elements of the submission, and a manufacturers’ wider HEOR evidence generation strategy as possible. That is, a single study could be used to generate data to inform disease incidence/prevalence, current treatment patterns, outcomes occurring under current standard of care and resource use associated with current standard of care.

Limitations of primary care databases should be considered in planning for the use of these databases to inform a NICE submission. Data quality is an issue with primary care databases as it is with most other real-world databases. Since most real-world data is not collected for research purposes, it can suffer from missing data and bias as a result of primary care being episodic and reactive [16, 17]. As a result of these issues it is often common for the investigator to have to clean the data using statistically rigorous and valid methods and to consider specific study designs to overcome a number of the data issues encountered [16]. An additional consideration in addressing data issues is that of linkage with additional data sources such as HES and disease registries. Linkages can provide broad datasets capable of capturing healthcare attendances across a range of settings and more detailed clinical data on certain diseases, they should therefore be considered when studying any condition not exclusively managed in primary care. As highlighted above, data on prescribing of drugs in secondary and tertiary settings is not typically available through routine linkages with primary care databases therefore these databases may not be suitable when such data are important to the research question. It should also be noted that primary care databases alone cannot typically be used to inform clinical effectiveness, since at the time of submission, there is likely to be no data available for new technologies in order to compare the relative effectiveness to current treatments. Given concerns regarding unmeasured confounding, in the rare cases where relative effectiveness estimates are available from primary care databases, they are unlikely to be accepted by NICE and other Health Technology Assessment (HTA) bodies as anything more than supportive evidence.

There are also limitations to this study, one of which is the ability to identify NICE TA submissions that have been withdrawn. Documents on these TAs are generally not available and therefore were not included in the analysis when identified. This study may therefore underestimate the usage of RWE from primary care databases in NICE TA submissions. Further to this point, the search strategy used will not retrieve any documents where the keywords or phrases used in our searches are not included in the TA and its associated documents, or where the documents have not been indexed by the search engine used. However we believe it unlikely that there are many TA submissions in which one of the databases has been used and the name of the database has not been mentioned somewhere in the commentary or references of the TA documents; particularly given that reporting guidelines for such studies recommend including the name of the database in the title of the article [18]. Additionally, there may be some TA submissions in more recent years that have been submitted that have yet to be published by NICE. Despite these limitations, we believe that our search strategy was relatively exhaustive as our use of the Google search engine allowed us to capture instances in which the use of the primary care database was mentioned in pdf documents associated with the TA but not in the guidance itself. This is an improvement on previous studies using searches of bibliographic databases and the search facility on the NICE website which captured a much small number of TAs [19, 20]. As mentioned in the introduction, primary care databases are commonly linked with additional databases and registries to augment the data contained in them. It is possible that that linked studies are better received by the ERG and committee than unlinked studies however we have not explored the impact of linkage in this study due to concerns that reporting of whether the primary care database was linked or not may be incomplete in NICE documents.

A short summary of this work was presented at ISPOR Europe 2019 [21].

## Conclusions

Studies that were conducted for purpose were much more positively received by the ERGs and appraisal committees than studies that were repurposed. Comments were more likely to be received when evidence from primary care databases was used to inform the CE model.

In conclusion, the potential of UK primary care databases in NICE submissions is increasingly being realised, particularly in informing the parameters of economic models. The use of the databases should therefore be given greater consideration when planning HEOR strategies to support market access in England.

## Supplementary information

**Additional file 1.** Breakdown of disease areas where primary care databases are used in NICE technology appraisals.

**Additional file 2.** A list of all the NICE technology appraisals reference numbers that were included in the analysis.

## Data Availability

The datasets generated and/or analysed during the current study are not publicly available as they are the proprietary data of the funder of this study, however they are available from the corresponding author upon request. All materials that fed into the generated dataset are available from the NICE website (https://www.nice.org.uk/) and a list of the relevant TAs contributing to the analysis are provided as a supplementary file.

## Abbreviations

HTA: Health technology assessment
RWE: Real-world evidence
CPRD: Clinical Practice Research Datalink
GPRD: General Practice Research Database
RCT: Randomised controlled trial
THIN: The Health Improvement Network
TA: Technology assessment
NICE: National Institute for Health and Care Excellence
ERG: Evidence review group
CE: Cost effectiveness
PRISMA: Preferred Reporting Items for Systematic Reviews and Meta-Analyses
BNF: British National Formulary
HEOR: Health economics and outcomes research
EHRs: Electronic health records
